# Understanding and caring for an indirect ophthalmoscope

**Published:** 2017-02-10

**Authors:** Ismael Cordero

**Affiliations:** 1 Clinical Engineer, Philadelphia, USA. **ismaelcordero@me.com**

The binocular indirect ophthalmoscope, or indirect ophthalmoscope, is an optical instrument worn on the examiner's head, and sometimes attached to spectacles, that is used to inspect the fundus or back of the eye. It produces an stereoscopic image with between 2x and 5x magnification. It is valuable for diagnosis and treatment of retinal tears, holes, and detachments. The pupils must be fully dilated for it to work well.

In a dark room, the examiner orientates his/her head so that light from the internal light source is directed into the patient's eye. A positive-powered condensing lens is held by the examiner at its focal length from the patient's eye, serving two purposes (Figure 1):

1. The lens ‘condenses’ light from the illumination system towards the patient's pupil.
2. Light reflected from the retina passes back through the lens creating a real, horizontally and laterally inverted image of the fundus situated between the lens and the examiner.

The viewing system of the instrument (Figure 2) consists of a pair of low-powered convex lenses. This design affords the examiner a stereoscopic view of the virtual image. The +20D lens is the standard lens for general examination offering 3x magnification and a field of view of approximately 45°. A +30D lens will offer 2x magnification along with a field of approximately 65°. These higher powered lenses are commonly used to examine small children and those with small pupils. They can be thought of as more forgiving than the lower-powered lenses, and as such are often advocated as a good choice of lens for those new to the indirect ophthalmoscope.

**Figure 1.**
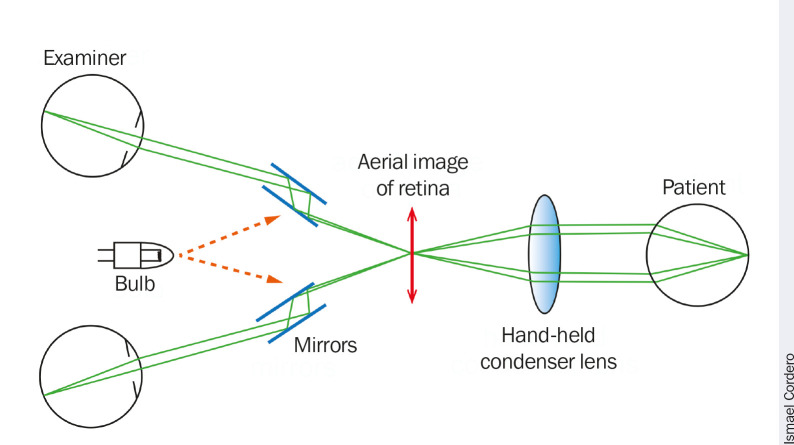
How an indirect ophthalmoscope works

Indirect ophthalmoscopes use halogen bulbs as the light source although many newer models use LED light sources which operate much cooler and last much longer. The newer models may incorporate battery packs that can be worn on the examiner's belt or can even be incorporated into the headband itself. These make it possible to use the indirect ophthalmoscope without the movement restrictions caused by power cables.

**Figure 2.**
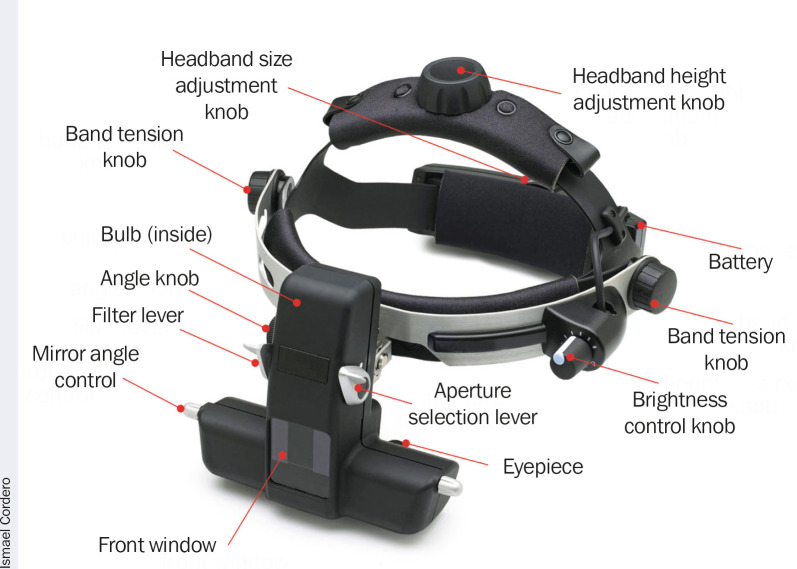
Indirect ophthalmoscope viewing system

The indirect ophthalmoscope offers some advantages over the direct ophthalmoscope:

- It permits binocular vision with depth perception (stereoscopic vision).
- It has a wider field of view.
- It can be combined with scierai indentation to examine the anterior retina.
- It is not affected by the refractive state of the patient’ eye.
- It may be used in the operating room without contamination.
- It accommodates a larger and brighter light source, which permits the examiner to penetrate moderate cataracts and to see more retinal detail.

## Care

- Keep the instrument in its case when not in use.
- Make sure the on-off switch is fully turned off (a click sound will be heard) before placing the instrument in its case.
- Recharge the batteries at the end of each work day.
- Wipe the headband and the instrument surfaces with a cloth dampened in mild disinfectant every day.
- Clean the lens by using hard contact lens cleaner and warm water and then drying it with a soft, lint-free cloth.
- If needed, sterilise the condensing lens by placing the lens in a cidex solution for 5–10 minutes, by ethylene oxide sterilisation, or by placing it in a formalin chamber. You can also autoclave the lens in a steel chamber with perforation for steam.

